# Energy landscapes direct the movement preferences of elephants

**DOI:** 10.1111/1365-2656.70023

**Published:** 2025-03-25

**Authors:** Emilio Berti, Benjamin Rosenbaum, Fritz Vollrath

**Affiliations:** ^1^ EcoNetLab, German Centre for Integrative Biodiversity Research (iDiv) Halle‐Jena‐Leipzig Leipzig Germany; ^2^ Institute of Biodiversity Friedrich‐Schiller‐University Jena Jena Germany; ^3^ Department of Biology University of Oxford Oxford UK; ^4^ Save the Elephants Nairobi Kenya

**Keywords:** elephant, energy landscape, habitat preference, *Loxodonta africana*, movement, step selection

## Abstract

1. The movements of animals affect biodiversity, ecological processes, and the resilience of an ecosystem. Movements carry both costs and benefits, and the use of a given landscape provides important insights into an animal's behavioural ecology and decision processes, as well as elucidating ecosystem complexity and informing conservation measures that are ever more important in the age of rapid global changes.
2. The mobility and habitat preferences of African savanna elephants (*Loxodonta africana*) offer a good example to explore the concept of ‘energy landscapes’, that is, the interplay between the cost of locomotion and vegetation productivity, balanced by topography, availability of water and human presence and pressures.
3. Our results, building on tracking data from 157 individuals collected between 1998 and 2020 in Northern Kenya, show that energy landscapes explained the elephants' usage of the landscape. In particular, we found that individuals generally avoided energetically costly areas and preferred highly productive habitats. We also found that water availability is important in determining habitat usage, but that its effect varied greatly among elephants, with some individuals preferring habitats avoided by others.
4. Our analysis highlights the importance of the energy landscape as a key driver of habitat preferences of elephants. Energy landscapes rely on fundamental biomechanical and physical principles and provide a mechanistic understanding of the observed preference patterns, allowing us to disentangle key causal drivers of an animal's preferences from correlational effects. This, in turn, has important implications for assessing and planning conservation and restoration measures, such as dispersal corridors, by explicitly accounting for the energy costs of moving.

## Graphical abstract

Energy landscapes explain the movement behaviour of elephants in Northern Kenya: they avoid energetically costly areas and preferred highly productive habitats. This highlights the importance of the energy landscape as a key driver of habitat preferences of elephants, with important implications for assessing and planning conservation and restoration measures.

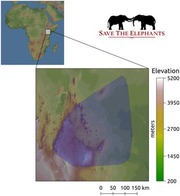

## 1 INTRODUCTION

Animal movement maintains important ecosystem functions such as seed and nutrient dispersal (Doughty et al., 2013; Guimarães et al., 2008); it promotes ecosystem stability (Gravel et al., 2016) and fosters biodiversity (Wilson, 1992). Important players for these processes are megafauna, that is, animals ≥45 kg (Martin & Klein, 1989), which have large home ranges (Kelt & Van Vuren, 2001) and can thus disperse nutrients, seeds, and energy across large areas (Doughty et al., 2013; Guimarães et al., 2008; Malhi et al., 2016). Megafauna were once widespread globally before human pressure triggered their extinction and restricted their distribution (Sandom et al., 2014) and home ranges (Hirt et al., 2021), with severe effects on overall biotic connectivity (Berti & Svenning, 2020). In particular, of the 48 megaherbivores (≥1000 kg) present globally at the beginning of the Late Pleistocene (starting around 120,000 years ago), only 8 have survived until today, almost all at risk of extinction and with decreasing distribution ranges (IUCN, 2022). In light of a new ongoing mass extinction and pressing challenges due to climate change, it is paramount to conserve these remaining megafaunal animals as well as preserve their ecosystems.

The African savanna elephant (*Loxodonta africana*) is the largest megaherbivore alive today. Once spread across all of Africa, *L. africana* has today a fragmented distribution, with wild populations often constrained to protected areas (Edwards et al., 2024; Wall et al., 2021). Moreover, wild elephant populations show overall decreasing trends in numbers, mostly due to poaching and increased human land use (Chase et al., 2016; Ripple et al., 2015; Veldhuis et al., 2019). Therefore, fully understanding the habitat requirements of elephants is key to optimising conservation and restoration efforts to protect currently threatened elephant populations. Elephant behaviour is strongly controlled by the availability of resources, and they are bulk feeders with a generalist diet, poor digestive efficiency and high basal metabolic costs. Importantly, due to their enormous size, elephants incur substantial costs of locomotion, which can prevent them from accessing some habitats with high plant productivity (Wall et al., 2006). Energy landscape analysis, that is, elucidating the interplay between resource availability and locomotion costs, can thus provide important insights into elephant use of habitats (Wilson et al., 2012).

Recent studies have begun to outline and clarify the habitat preferences of elephants and describe the drivers of their patterns in movement behaviour. Partly to account for the cost of locomotion, elevation is commonly used as a predictor for habitat preferences of elephants (Asner et al., 2016; Chibeya et al., 2021; Ngene et al., 2009; Talukdar et al., 2020), usually explaining a large proportion of an elephant's behaviour. However, energy cost of locomotion is not related to elevation, but to slope, that is, the incline of the terrain and in a strongly non‐linear way (Pontzer, 2016). Additionally, as other factors covary with elevation, it is not clear whether elevation itself influences elephants' movements rather than being a convenient proxy to capture other abiotic and biotic processes. For instance, vegetation structure and water availability, as well as human presence and partial pressure of oxygen, all vary with elevation, which may thus shape movement behaviour only indirectly, for example, by affecting soil and water dynamics, vegetation structure and anthropogenic pressure (Asner et al., 2016; Chibeya et al., 2021; Ngene et al., 2009; Taher et al., 2021; Talukdar et al., 2020). This partly hinders efforts to fully comprehend the habitat preferences of elephants, for example, by masking the real causal associations between the environment and habitat preferences with spurious correlations. Moreover, a number of observations suggest that elephants may not be as much limited by elevation as commonly thought, with some recorded cases of elephants climbing ~2000 m in elevation (Choudhury, 1999; Kuswanda et al., 2022).

In this study, we used the recently published analytical tool and method *ENERSCAPE* (Berti et al., 2022) to estimate the energetic costs that an animal has to sustain in order to travel across a topographically explicit landscape (Shepard et al., 2013; Wall et al., 2006). Enerscape estimates the cost of locomotion for legged terrestrial animals using the body mass of the individuals and the incline of the terrain obtained from elevation data. Specifically, we used enerscape to investigate how energy costs of travel influence the movement decisions and thus habitat preferences of 157 elephants in the Samburu area of Northern Kenya. This approach takes into account both the body mass of the animal and the slope of the terrain traversed (Berti et al., 2022; Pontzer, 2016). It assigns lower costs for smaller animals and slopes close to −8 degrees and thus captures the mechanistic cause of habitat preferences owing to the costs of locomotion better than simply using elevation as a topological parameter (see also Wall et al., 2006).

We assessed whether elephants respond to energy landscapes determined by locomotion costs and resource availability. In particular, we tested whether: (H1) elephants avoided areas with high costs of locomotion; and (H2) elephants preferred areas with high vegetation productivity. Additionally, because the cost of locomotion may increase with movement speed (Wilson et al., 2021), we also tested whether (H3) elephants avoided highly costly areas particularly when travelling at high speeds. If these hypotheses are supported (i.e. not falsified) by our analysis, then there is strong support for using energetic costs and gains in energy landscape approaches for understanding the habitat preferences of elephants. We achieved this by analysing GPS telemetry data using a step‐selection function to understand which environmental factors influenced the habitat preferences of elephants. By explicitly testing these causal relationships, our study aims to elucidate the direct drivers of elephants' preferences, providing deeper insights into possible mechanisms determining habitat use, thus helping to generate specific hypotheses testable with fine‐grained data.

## 2 MATERIALS AND METHODS

We estimated energy landscapes by calculating the energy costs of locomotion and vegetation productivity, that is, the potential energy gains extracted from resources. We tested our hypotheses by estimating the direct effects of these two components of the energy landscape on elephant preferences using a step‐selection function approach. As human pressure and water availability, which covary with elevation (Chibeya et al., 2021; Ngene et al., 2009), influence the movement of elephants (Chibeya et al., 2021; Sach et al., 2019; Taher et al., 2021; Talukdar et al., 2020; Wall et al., 2013), we also considered elevation, distance to human settlements, and to permanent water bodies as explanatory variables for elephant preferences.

### 2.1 GPS collar data

**FIGURE 1:**
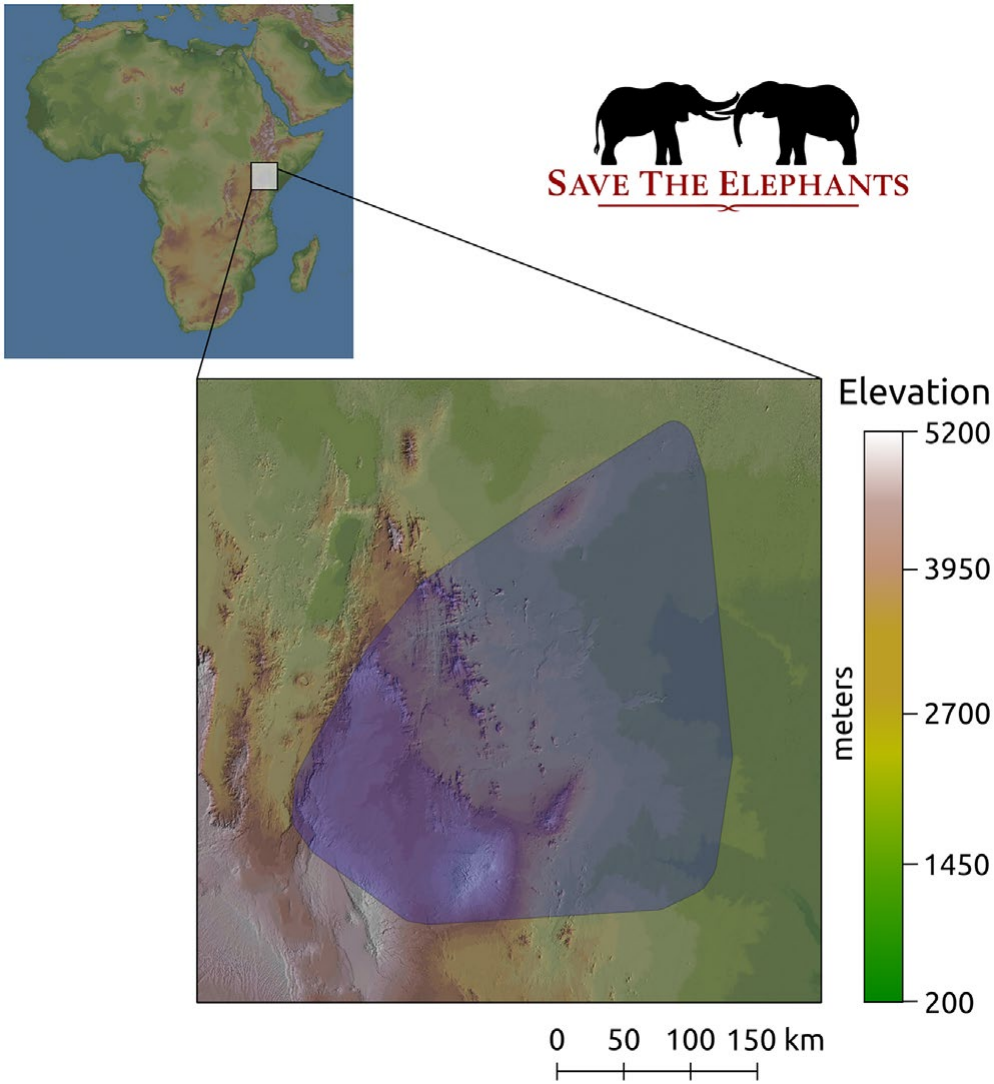
The study region in Northern Kenya (Isiolo, Laikipia, Marsabit, Meru, and Samburu counties). Terrain colours show the elevation of the terrain. The blue shade is the minimum convex polygon containing all GPS records for the elephants. GPS data was made available by *Save The Elephants*.

GPS data for 172 elephants spanning the period 1998–2020 were made available by Save The Elephants (STE) and its team of researchers and monitors. STE is a non‐profit organization that, in collaboration with the Kenyan Wildlife Service and Kenya's Wildlife Research and Training Institute, promotes the protection of elephants and related ecological research with, among others, a multi‐decades GPS radio tracking project in Kenya. We were given access to relevant elephant telemetry data for the Samburu region in Northern Kenya (36–39° E, −0.36–2.81° N; Figure 1). This area has a large elevational gradient (from ~200 to ~5000 m a.s.l.), with rainfall mostly concentrated in two periods (April–June and October–December) and strongly influenced by the presence of mountain peaks. This variation in altitude and rainfall across the landscape is associated with changes in land use: forested areas can be found at high elevations, whereas at lower elevations the landscape is dominated by savannah, with interspersed agricultural and farming areas. GPS data were already processed by STE to assure the quality of the records, that is, GPS fixes that had inaccurate longitude and latitude coordinates were already removed. To ensure that subsequent GPS fixes were regularly spaced in time, that is, all elephants had constant sampling frequency, we resampled the original GPS data to one fix per hour (±6 min). When a fix was separated by more than 66 min from the previous, we kept it, but considered it and subsequent fixes as part of another, separate track for the same animal. These tracks were then analysed as separate observations for the same individual (see below). This step was necessary to make sure that the movement was modelled consistently across the whole time span of the recordings for each individual, for example, the distance travelled between fixes was comparable.

### 2.2 Remote sensing data and energy landscapes

We calculated the distance to human settlements using the World Settlement Footprint product (Marconcini et al., 2020) and the distance from permanent water bodies using the ESA WorldCover product (Zanaga et al., 2021). Both distances were calculated as the great‐circle distances with a precision of 1 m. A digital elevation model (DEM) for the region of interest at a resolution of 30 × 30 m was obtained from NASADEM (NASA JPL, 2020). Energy landscapes are defined as the energy costs of travelling across a landscape offset by the energy gains obtained from the resources (Shepard et al., 2013; Wilson et al., 2012). Because of a lack of knowledge on how to integrate the two into one metric that is meaningful for elephants, we kept the cost of locomotion and vegetation productivity as separate layers in our analyses. Costs of locomotion were computed using the R package enerscape (Berti et al., 2022), which calculates the energy cost of travel across the landscape using the body mass of the animal and the slope of the terrain traversed (Pontzer, 2016). The model used by enerscape has been shown to scale well across species and for a large range of body masses (from 0.78 g to 4000 kg; Berti et al., 2022) and explains most of the variation in the cost of locomotion for terrestrial legged animals (*R* ^2^ = 0.95; from Pontzer, 2016). Because the cost of locomotion depends on the body mass of animals, we calculated energy landscapes for females and males separately. As GPS collars were mounted only on adult individuals, we assumed a body mass of 2744 kg for females and 6029 kg for males, which are typical values for adult individuals (Laws & Parker, 1968). These values represent average values for the populations and were used because we did not have body mass information for each elephant.

As a proxy for vegetation productivity, we used the Normalized Difference Vegetation Index (NDVI), calculated from the Sentinel‐2 Copernicus mission (Harmonized Sentinel‐2 MSI, Level‐2A; https://www.esa.int/Copernicus/Sentinel‐2/Data_products). NDVI, which has values from −1 to 1, is a measure of the relative abundance of chlorophyll and was used here as a proxy for plant productivity. We calculated the median monthly NDVI using all images spanning the whole period of the Sentinel‐2 mission (from June 2015 to February 2023). First, we removed pixels that were identified as clouds of cirrus formations as well as all images that had less than 20% of their area with clear sky conditions. Then, we split the dataset into calendar months and calculated the median reflectance of the near‐infrared (*NIR*) and red (*RED*) bands, representing the monthly median values of the bands across the whole time period 2015–2023. Finally, we calculated NDVI, for each month separately, as: $\text{NDVI}=\frac{\mathrm{NIR}-\mathrm{RED}}{\mathrm{NIR}+\mathrm{RED}}$. As the satellite data did not span the whole temporal range of the GPS data, we assumed that the values obtained for the period 2015–2023 were representative also of the previous years. In other words, we used a monthly NDVI metric that reflected the overall value for the last 8 years and assumed that the previous years had similar overall monthly trends, which were largely consistent for the period 2001–2022 for data obtained from MODIS at a coarser resolution of 500 × 500 m (Figure S1).

### 2.3 Fitting hidden Markov models (HMMs)

To define a redistribution kernel, that is, a probabilistic moving range that reflects the observed movement patterns of individuals, as required by the step‐selection function (Beumer et al., 2023), we decomposed the movement process into distinct underlying states using a Hidden Markov Model approach (HMM). HMMs are a class of state‐space models that describe animal behaviour as a set of states defined by movement parameters and by the probabilities of transitions among states (Jonsen et al., 2005; McClintock et al., 2020). Each state is characterized by its movement parameters, for example, states associated with long‐distance dispersal have higher average step lengths. HMMs were fitted to each elephant individual separately. Therefore, their movement states and relocation parameters are not quantitatively comparable. We stress that (i) we use HMMs to sample unused locations in the landscape to perform the step‐selection function and that (ii) we are not focusing here on their interpretation. What we take from the HMMs are the movement parameters that describe the redistribution kernel.

To fit HMMs, we calculated, from the GPS fixes, the relocation step length (meters) and the turning angle (radians). Step lengths were assumed to follow a Gamma distribution, characterized by two parameters: the mean and standard deviation. Turning angles were assumed to follow a Von Mises (VM) distribution, characterized by two parameters: the mean turning angle and the concentration of the distribution around it. If an individual had non‐contiguous fixes, as obtained from our resampling method, we fitted the whole GPS data together, but specified different tracks to be considered as separate observations. Importantly, different tracks had the same sampling frequency and did not violate common assumptions of HMMs (Michelot et al., 2016). In other words, we assumed that the individual moved according to some generic behaviour decisions that did not change across tracks, while ensuring that non‐contiguous fixes did not introduce any biases into the fitting procedure. Tracks of less than 20 relocations were deemed to have too few data points and were removed; therefore, we excluded two individuals from analyses with only 15 and 63 total relocation data points.

Fitting HMMs requires a pre‐defined number of movement states and initial distribution parameters. This may influence HMM results, as different numbers of states can lead to different parameter estimates, and changing the starting parameters can lead to different fitted estimates (Michelot et al., 2016). To explore these potential issues, we fitted several HMMs per individual, changing the number of behavioural states and the starting parameters, and assessed the consensus of different runs for the fitted parameters. Specifically, we fitted three sets of models, each set with one, two, or three movement states, with the three‐state model being the one preferred in the majority of previous studies (Polansky et al., 2015; Taylor et al., 2022). For each set, we then fitted 10 model replicates that differed in their initial parametrization to assess the sensitivity of results to initial starting conditions. The starting parameters were randomly drawn from a uniform distribution bound to the 10%–90% quantiles of the observed movement values from GPS recordings; for HMMs with more than one state, this range was additionally divided into corresponding intervals. For instance, the three starting parameters for the three‐state models were sampled from uniform distributions U(q_10%_, q_40%_), U(q_40%_, q_70%_), U(q_70%_, q_90%_), respectively.

We then compared HMMs within replicates using AIC and selected the most parsimonious model. We retained only the individuals for which all parsimonious models had the same number of movement states, indicating a fair amount of consensus among HMM runs; six individuals were thus removed from further analyses. A total of 164 individuals were thus used in the step selection function analysis (Table S1). From the fitted HMMs, we also assigned to each GPS location the most likely movement state, obtained using the Viterbi algorithm (Zucchini & MacDonald, 2009). HMMs were fitted using the R package moveHMM (Michelot et al., 2016).

### 2.4 Step selection function

To assess the habitat preferences of elephants, we used a step‐selection function approach (SSF). SSFs are particularly suited to analyse our dataset as they take into account the movement pattern of the individuals and the serial structure of GPS data (Thurfjell et al., 2014). SSFs contrast used and unused locations, the latter being sampled in our approach using probabilistic kernels defined by the parameters of the movement state as obtained from the HMMs (Karelus et al., 2019). Using the results of HMMs, we: (1) assigned a movement state to each GPS location using the Viterbi algorithm (Zucchini & MacDonald, 2009); (2) sampled step length and turning angle from their predictive distributions; and (3) obtained unused locations for the next step by calculating the displacement from the observed GPS location. This allowed the availability domain of the habitat to change depending on the animal state, which has been shown to reduce common inference pitfalls in SSFs (Beumer et al., 2023). In particular, if ${\left(x,y\right)}_{t}^{1}$ is a GPS location of an individual at time *t* in movement state *s*, and $\alpha_{t}=\text{atan}\left(\frac{y_{t}-y_{t-1}}{x_{t}-x_{t-1}}\right)$ is the angle of the direction of the movement, we obtained an absence for the next step by sampling the step length (l) and turning angle (θ) from their respective distributions ($l_{t}∼{\text{Gamma}}_{s}$ and $\theta_{t}∼{\mathrm{VM}}_{s}$) and by adding this displacement to the GPS fix: ${\left(x,y\right)}_{t+1}^{0}={\left(x_{t}^{1}+l_{t}\cdot \cos\left(\alpha_{t}+\theta_{t}\right),y_{t}^{1}+l_{t}\cdot \sin\left(\alpha_{t}+\theta_{t}\right)\right)}_{t}^{1}$. Following previous recommendations (Karelus et al., 2019; Thurfjell et al., 2014), we sampled only six unused steps for each GPS observation as this number is likely enough to accurately fit SSFs, which are robust to different choices of numbers of unused steps, while reducing computational costs.

Using the used and unused steps, the latter sampled using the redistribution kernels obtained from HMMs, we estimated the habitat preferences of elephants by fitting a conditional logistic regression for each individual separately. We specified the step ID as strata, thus contrasting the environmental factors of the GPS locations with those of the sampled absence locations. As covariates, we considered elevation (m), cost of locomotion (kcal), NDVI (adimensional), elevation (m), the distance (m) to the closest permanent water body, and the distance to the closest human settlement. Since previous studies suggested that elevation may indirectly affect preferences by influencing the other covariates (Asner et al., 2016; Berti et al., 2022; Chibeya et al., 2021; Ngene et al., 2009; Taher et al., 2021; Talukdar et al., 2020), we assessed collinearity among predictors using the Pearson correlation coefficient and retained only the variables that showed low correlation, namely with a correlation coefficient <0.70 (Dormann et al., 2013).

To test whether the cost of locomotion (H1) and vegetation productivity (H2), i.e. the energy landscape, affected consistently elephants' preferences, we obtained the point estimates as well as the 95% confidence interval for all covariates included in the final models. We thus assessed how many elephants avoided areas with high costs of locomotion and preferred areas with high NDVI values. We also obtained, similarly to Beumer et al. (2023), an estimate of the population‐level preferences for each predictor by calculating the weighted average and standard error across all individuals, using as weights the reciprocal of the standard error of the estimates as evaluated in the SSF. We calculated the p‐value of these population‐level estimates using the *pt*() function in R. Additionally, we tested whether elephants showed a more pronounced selection against areas with high energy costs and in favour of highly productive areas when moving at higher speeds (H3). This was achieved by filtering the data in order to retain only the steps specific to each state and fitting again the conditional logistic regression, which allowed us to assess whether the habitat preferences of the elephants differed among movement behaviour decisions. Because seven individuals had negligible variation in the cost of locomotion across the studied area, we excluded these individuals from the results. All predictors were centred to have zero mean and scaled to have unit variance before fitting the conditional logistic regressions (Table S4).

Analyses were performed using the R and python programming languages. State‐space modelling (HMMs) and step‐selection functions were performed in the UTM37N coordinate reference system (+proj = utm + zone = 37 + a = 6378249.145 + rf = 293.465 + towgs84 = −157,‐2,‐299,0,0,0,0 + units = m + no_defs) at a resolution of 30 × 30 m. Conditional logistic regressions were performed using the package survival (Therneau & Lumley, 2015). Weighted statistics were calculated using the functions wtd.mean() and wtd.var() from the *Hmisc* package (Harrell Jr. & Harrell Jr., 2019).

This study did not require ethical approval.

## 3 RESULTS

The best state‐space models (HMMs), as assessed using AIC, always had three behavioural states, as also suggested by previous studies (Polansky et al., 2015; Taylor et al., 2022). The 10 replicates of HMM per individual had a high degree of consensus (Figure S2), indicating (i) that HMM replicates for the individuals converged to comparable, if not identical, values and (ii) that fitted parameters for the movement distributions were reliable. The first state was characterized by a slow, undirected movement (average step length = 89 m; angle concentration = 0.23), the second state by intermediate speeds and more directed movement (average step length = 363 m; angle concentration = 1.30), and the third state by a fast, directed movement (average step length = 1100 m; angle concentration = 2.14). Because HMMs were fitted to the elephant individual separately, these states are only qualitatively comparable among individuals (e.g. the slow states of individual A and B are slower compared to their respective faster states), but not quantitatively so (e.g. the slow state of individual A may be faster than the slow state of individual B). Moreover, the average values reported above had large standard deviations (Table S2), indicating a large inter‐individual variability in the movement patterns and in the redistribution kernels and movement behaviours. Because of these limitations, we did not interpret the states from an ecological perspective.

Cost of locomotion was strongly correlated with elevation for only one of the 157 elephants (Pearson *ρ* = 0.71), whereas for the other 156 individuals the correlation was generally low (*ρ*_mean_ = 0.18; Figure S3). Additionally, the correlation between NDVI and elevation was generally low (*ρ*_mean_ = 0.17) and only two of the 157 individuals had a coefficient above 0.70. NDVI and cost of locomotion were very weakly correlated (*ρ*_mean_ = 0.13), with the maximum correlation coefficient being 0.51. The lack of strong correlations between cost of locomotion and NDVI with elevation indicates that potential energy costs and gains are not interchangeable with elevation. We found, however, that distance to water bodies was strongly correlated with elevation for 19 elephants and with distance to urban settlements for 39 individuals (Figure S3). Because including correlated predictors may bias coefficient estimates of our statistical models, we omitted from our statistical model any variables that had a Pearson correlation coefficient >0.70 for at least one individual. We thus selected cost of locomotion, NDVI, and distance to water bodies (*ρ*_max_ = 0.51) as the only predictors of elephants' preferences.

**FIGURE 2:**
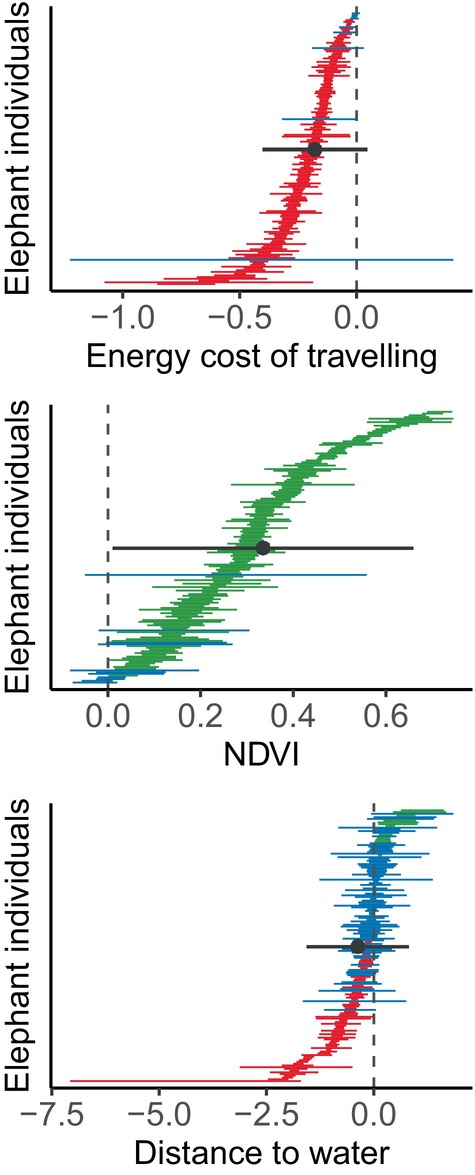
Coefficient estimates from step‐selection function. Horizontal bars show the 95% confidence interval of elephant preferences. Each bar represents one elephant individual. Colours show the effect of the estimate: Red indicates negative effect on preferences (*p* < 0.05), green a positive effect (*p* < 0.05), and blue no effect (*p* > 0.05). The circles show the population‐level estimates, obtained as the weighted average across all individuals, and the grey range the 95% confidence intervals.

Inspecting the coefficients estimates from the step‐selection function, we found that 148 of the 157 elephants avoided locations with high costs of locomotion (*p* < 0.05; Figure 2, Table S3), preferring instead to move in areas associated with low costs of transport. There were only nine elephants that did not show avoidance or preference for costs of locomotion (*p* > 0.05) and no elephants preferred to move to areas associated with high costs. The population‐level selection coefficient, obtained as the weighted average across all individuals, was −0.18 (SE = 0.11; 95% CI = [−0.40, 0.06]), indicating that the population, as a whole, avoided costly areas, even if this observed pattern is marginally not significant at *p* = 0.06 (*t* = −1.56, df = 156). These results confirmed our first hypothesis, i.e. that elephants strongly avoided areas with high costs of locomotion while reconfirming that there was substantial variation across individuals. In support of our second hypothesis, we also found that elephants consistently preferred habitats with high NDVI values. In particular, 146 elephants preferred areas with high NDVI (*p* < 0.05), with only 11 individuals showing no preferences (*p* > 0.05). The population‐level selection coefficient for NDVI was 0.34 (SE = 0.17; 95% CI = [0.01, 0.66]), indicating the population moved towards habitats with abundant resources. Individuals showed large variation in their individual behaviour also for NDVI, as shown by the weighted average, which is significant, but only marginally so (*p* = 0.02; *t* = 2.02, df = 156). We also found that elephants responded to distance to water bodies, but less consistently compared to costs of locomotion and NDVI. In particular, 64 individuals avoided areas that were distant from water (*p* < 0.05), 13 preferred habitats far from water (*p* < 0.05), and 80 individuals did not show any preference (*p* > 0.05), highlighting a high heterogeneity among individual preferences when considering distance to water as a predictor of elephant habitat preferences. The population‐level selection coefficient for distance to water was −0.37 (SE = 0.61; 95% CI = [−1.56, 0.82]), indicating the population avoided moving far away from water bodies albeit with a very high variability in individual preferences and without a clear overall population‐level pattern (*p* = 0.27; *t* = −0.61, df = 156). Overall, these results confirmed our two main hypotheses: elephants responded to energy landscapes consistently, avoiding areas with high costs of locomotion and preferring areas with high vegetation productivity.

**FIGURE 3:**
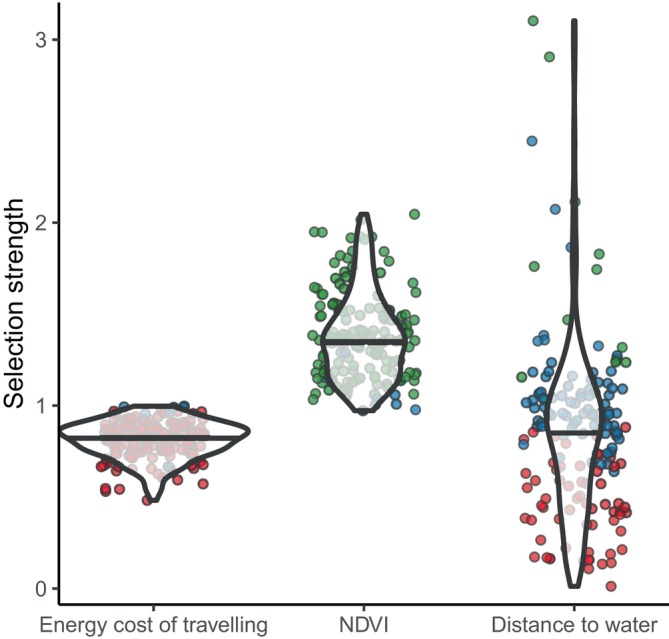
Selection strength of the factors influencing elephant preferences. Points show the selection strength of the three studied factors for each elephant, with colours indicating a negative effect on preferences (red), a positive effect (green), or no effect (blue). The violin plots show the distribution of the strength coefficients, with black horizontal line showing the median value.

When inspecting the strength of the selection across all individuals (Figure 3), the probability of taking a step increases by, on average, 37% (SD = 24%) for an increase of one standard deviation in NDVI. For the cost of travelling and distance to water, instead, this probability decreased. Specifically, it decreases by 19% (SD = 10%) for an increase of one standard deviation in energy costs and by 14% (SD = 48%) for an increase of one standard deviation in distance to water.

**FIGURE 4:**
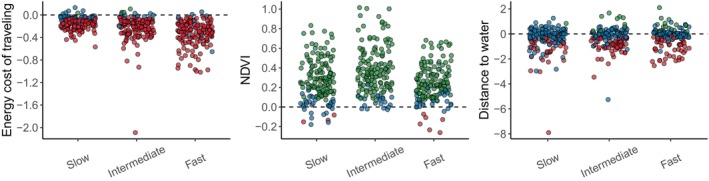
Coefficient estimates for the step‐selection function for the three movement states: Slow, intermediate speeds, and fast. Each circle represent an elephant individual, with colours showing the effect of the estimate: Red indicates negative effect on preferences (*p* < 0.05), green a positive effect (*p* < 0.05), and blue no effect (*p* > 0.05).

When analysing the preferences for the three movement states separately, we found generally similar trends to the overall preferences (Figure 4). In particular, individuals generally avoided high costs of locomotion and preferred highly productive habitats but showed contrasting trends among individuals when considering the preferences for distance to water. Corroborating H3, individuals showed a stronger avoidance of high costs of locomotion when they were moving faster: 116 (74%) individuals avoided costly areas when moving slowly, which increased to 136 (87%) when moving at intermediate speeds and to 146 (93%) when moving fast. Moreover, individuals preferred high NDVI areas especially when moving at intermediate speeds (*n* = 136; 86%) compared to both moving slowly (*n* = 119; 76%) and fast (*n* = 121; 77%). For distance to water, 36 individuals avoided areas far from water when moving slowly (23%), which increased to 53 (34%) at intermediate speeds and to 49 (31%) when moving fast, although the majority of elephants did not show any preferences. These results suggest that elephants adjust their behaviour depending on their movement/behavioural state. In particular, elephants avoided costly areas when moving fast but tended to show less strong preferences when moving slowly. Despite this, the large majority of the individuals still avoided such costly areas in all movement states.

## 4 DISCUSSION

We assessed the habitat preferences for 157 elephants from GPS recordings spanning around 21 years in the Samburu region in Kenya. Our analysis revealed that almost all elephants strongly selected favourable energy landscape areas, i.e., avoided areas with high movement costs while preferring habitats with high vegetation productivity. This corroborates and further specifies previously observed patterns showing that landscape topography seems to be a key driver of elephant behaviour in the ecosystem also studied by Wall et al. (2006). Moreover, our findings add to evidence showing that animals seem to respond to energy landscapes, for example, with birds using favourable wind uplift (Eisaguirre et al., 2020). Our analysis further shows that the other predictor analysed, that is, distances to water varied greatly among elephants, which suggests high individual variation concerning this specific preference. For instance, only 41% of the individuals preferred areas in proximity to water. This is a small proportion when compared to the percentages of elephants that showed preferences for areas with low costs of locomotion (94%) and high vegetation productivity (93%). This may be due to elephants exhibiting a functional response, that is, changing preferences depending on the ecological context (Holbrook et al., 2019), for water availability, but not for energy landscapes. The lack of preferences for water availability may be an artefact of our choice to remove from the analyses the covariates related to human pressure and elevation, which were highly correlated with water availability for some individuals. Of the two, human pressure may particularly modulate elephants' responses to water availability, as elephants may need to evaluate trade‐offs between where to find water and conflicts with humans.

Additionally, we analysed each elephant individually and summarised their preference responses using weighted averages, while noting that other methods exist to account for individual variability in step‐selection functions, for instance, the random effect framework of Muff et al. (2020). Importantly, our analysis does not negate the significance of predictors beyond the energy landscape in influencing the habitat usage of elephants. Rather, it highlights that the responses of individual elephants may be contingent upon the prevailing environmental conditions, such as seasonal variations, as well as their personal preferences. Individual water requirements, for example, might change not only for drinking but also for wetting the skin‐sponge to facilitate cooling during periods of high daily temperatures. Nevertheless, energy landscapes and in particular, costs of locomotion, which are based on fundamental biomechanical and physical principles, almost unequivocally explained preferences of elephants. Overall, our results highlight that the energy landscape is a key driver of habitat preferences for elephants and that it affects habitat usage consistently across individuals.

Contrary to previous studies that used elevation but not the cost of locomotion, our approach permits modeling explicitly a plausible causal relationship between terrain and movement preferences. Indeed, species do not respond directly to elevation but rather to other environmental factors regulated by elevation, most notably temperature and precipitation (Austin, 2002; Hof et al., 2012). This was the rationale commonly used in previous studies that used elevation as a proxy for unobserved environmental factors. The concept of energy landscapes is not new (Shepard et al., 2013; Wilson et al., 2012), not even for elephants (see Wall et al., 2006). However, dedicated software to calculate movement costs for terrestrial animals is a novel tool (Berti et al., 2022), which is now opening up the application of energy landscapes as a defining factor in studies of behavioural ecology in complex ecosystem landscapes.

Inclusion of elevation as a predictor variable may also, in addition to energy costs, serve as a proxy for other physical attributes of the terrain, such as slope or terrain ruggedness. This is because areas at higher elevations may possess steeper slopes and uneven terrains, which can influence the habitat selection of elephants. However, this is not always the case, and we suggest that using the cost of locomotion in an energy landscape framework, which joins biomechanical models with the physical aspect of the terrain and availability of resources, is a better approach due to the clear ecological assumptions and direct causal relationship that can be drawn between landscape and preferences.

It was rather surprising to discover that elephants did not show clear preference patterns for the distance to water bodies. Previous studies that analysed shorter time spans and that included seasonal variations found that precipitation patterns, indicating water availability, are important drivers of elephant preferences (Chibeya et al., 2021; Mulder et al., 2024; Sach et al., 2019; Taher et al., 2021; Talukdar et al., 2020; Wall et al., 2013). We acknowledge that discrepancies between these observations and our results may be due to the large spatial and temporal scale of our analysis, which focused on an area of around 40,000 km^2^ for a 21‐year timespan, omitting fine‐scale temporal variability such as seasonality in precipitation. For example, Bastille‐Rousseau et al. (2020) found that elephants prefer to stay close to permanent water bodies and human settlements during the dry season, but not in the wet season when water can be found also in temporary basins. In addition, because of the strong correlation among distance to water and distance to human settlements, and elevation, our estimates of the elephants' preference for habitat close to water may be partly biased.

Moreover, habitat preferences can be heterogeneous among elephant individuals (Bastille‐Rousseau et al., 2020; Bastille‐Rousseau & Wittemyer, 2022), which may further explain potential discrepancies between our results and previous studies. For instance, during the must‐reproductive period, bulls behave significantly differently from their normal behaviour (Taylor et al., 2020), while females with offspring may choose different habitats depending on the needs of the whole herd, rather than individual preferences.

Taken together, these considerations and new insights suggest future avenues of research in order to better understand and interpret the observed variability in preference among individuals as well as to disentangle further the causal relationships between environmental factors and the utilization of the landscape by elephants. Enerscape modelling allowed us to formulate hypotheses of elephant movement decisions and habitat use in a given landscape, in this case Samburu, at a given time. Taken forward, these hypotheses can be tested using more fine‐grained data, for example, GPS fixes obtained at higher frequencies coupled with terrain models at higher resolutions and environmental predictors measured concurrently to the telemetry data.

A particularly promising future direction would be to join the energy cost of transport with resource availability, which would allow modelling a more holistic energy landscape (Shepard et al., 2013). Here, we assessed the importance of these two factors of energy landscapes separately, that is, each cost of locomotion and vegetation productivity had its own coefficient estimates. By combining these two factors into a single metric that quantifies the net energy costs and gains of energy landscapes, future research could clarify additional details of elephant behaviour. Recent advances in integrating energy costs and gains into a step‐selection framework capable of jointly estimating animals' preferences and their redistribution patterns are promising (Eisaguirre et al., 2020; Klappstein et al., 2022). Importantly, such implementation could also account for seasonality in resource availability and define variable energy landscapes that change through time (Masello et al., 2017), improving the realism of our approach and its applicability to specific conservation issues (Bastille‐Rousseau & Wittemyer, 2021). Additionally, our findings that elephants show increasing preferences for reducing the costs of movement when travelling at higher speeds suggest that energy landscapes are particularly important during long‐distance, strongly directional movements. Importantly, as the travel speed of elephants is limited by high temperatures (Dyer et al., 2023), this approach could also explicitly model how habitat usage of elephants may change under climate change, with potentially extremely important insights for conservation efforts adapting to evolving environmental conditions.

Our work highlights the importance of energy landscapes to explain the habitat preferences of elephants. We expect this to be particularly relevant also to predict how elephants use the landscape both within their current distribution as well as for planning dispersal corridors for conservation and restoration planning. As the current distribution of both the African elephants (*Loxodonta africana* and *L. cyclotis*) and the Asian elephant (*Elephas maximus*) is fragmented (IUCN, 2022), we expect that in many ecosystems movement across isolated patches will be strongly influenced by energy landscapes. For example, corridors have been proposed to restore the fragmented distribution of elephants in Sumatra (Kuswanda et al., 2022); our study, and energy landscapes in general, could help in planning such corridors in areas that experience an energy landscape gradient. In addition to practical applications, our findings are also relevant for theoretical studies: as movement costs increase disproportionately with body mass (Pontzer, 2016), larger animals should be particularly affected by energy landscapes. This has implications for the dispersal of large animals and megafauna, which disproportionately enhance biotic connectivity and biodiversity (Berti & Svenning, 2020; Malhi et al., 2016), that should be further explored.

## 5 CONCLUSIONS

Our findings emphasize the importance of including energy landscapes as a key driver of habitat preferences for elephants and, potentially, for terrestrial megafauna in general. Using energy landscapes, instead of elevation, is theoretically supported by physical and biomechanical principles and has clearer ecological assumptions. This, we believe, is a strong argument for drawing a direct causal association between energy landscapes and preferences, as supported by our results. Our study should not be considered a definitive answer to explain animal preferences, but a path towards a more mechanistic understanding of why animals prefer certain habitats. With changing climate, most ecological parameters will change, and for large animals, even the metabolic costs of movement can increase because of overheating (Dyer et al., 2023). As we get better at collecting more fine‐grained data on ecological parameters, we need to develop more sophisticated models for their analysis, as we have shown here. This will provide a better understanding of how animals use the landscape and help conservation and restoration efforts in planning dispersal corridors to enhance recovery of isolated populations.

## AUTHOR CONTRIBUTIONS

Emilio Berti and Fritz Vollrath conceived the ideas; Emilio Berti and Benjamin Rosenbaum designed the methodology; Emilio Berti analysed the data and led the writing of the manuscript. All authors contributed critically to the drafts and gave final approval for publication. Our study does not include scientists based in the country where the study was carried out. We recognize that more could have been done to engage local scientists with our research as our project developed, and to embed our research within the national context and research priorities. We are planning to address these caveats in future research.

## CONFLICT OF INTEREST STATEMENT

We have no conflict of interest to disclose.

## Supporting information


**Table S1.** Number of elephants included in the study after each data processing step.
**Table S2.** Average values of the fitted parameters for the three states of the Hidden Markov Models.
**Table S3.** Number of elephants divided in statistical effect for the step‐selection function.
**Table S4.** Average and standard deviation of the variables included in the SFF across all elephants.
**Figure S1.** NDVI monthly trend for the period 2001‐2022 from MODIS.
**Figure S2.** Frequency distribution of the coefficient of variation of the Hidden Markov Models (HMMs) parameters.
**Figure S3.** Distribution of the Pearson's correlation coefficients between covariates among all elephants included in the study.

## Data Availability

Code and data available from Zenodo https://doi.org/10.5281/zenodo.14760726 (Berti et al., 2025). Due to the sensitive nature of the GPS data, we cannot share the original GPS data; queries to access it should be addressed directly to https://www.savetheelephants.org/.
