# Supplementary material for: Energy landscapes direct the movement preferences of elephants
Source: J Anim Ecol. 2025 Mar 25;94(5):908–18. doi: 10.1111/1365-2656.70023 (PMC12056349; doi:10.1111/1365-2656.70023)
Supplement: Supplementary file 1 — Table S1. Number of elephants included in the study after each data processing step. Table S2. Average values of the fitted parameters for the three states of the Hidden Markov Models. Table S3. Number of elephants divided in statistical effect for the step‐selection function. Table S4. Average and standard deviation of the variables included in the SFF across all elephants. Figure S1. NDVI monthly trend for the period 2001‐2022 from MODIS. Figure S2. Frequency distribution of the coefficient of variation of the Hidden Markov Models (HMMs) parameters. Figure S3. Distribution of the Pearson's correlation coefficients between covariates among all elephants included in the study. [file JANE-94-908-s001.pdf]

# Supporting information

**Table S1: Number of elephants included in the study after each data processing step.**

| | Original | enough fixes | HMMs with same number of states | significant energyscape range |
|---|---|---|---|---|
| Number of individuals | 172 | 170 | 164 | 157 |

**Table S2: Average values of the fitted parameters for the three states of the Hidden Markov Models.**

| Distribution | parameter | slow | intermediate | fast |
|---|---|---|---|---|
| gamma | mean (m / hour) | 90 (10–1666) | 351 (134–3704) | 1122 (370–4418) |
| gamma | standard deviation (m / hour) | 82 (6–999) | 231 (100–1805) | 625 (253–3262) |
| Von Mises | mean (rad) | -0.06 (-3.08–3.10) | -0.01 (-2.04–0.20) | 0.03 (-0.52–3.00) |
| Von Mises | concentration | 0.23 (0.01–2.10) | 1.28 (0.26–3.20) | 2.12 (0.68–5.80) |

The values between parentheses show full range of the estimates.

**Table S3: Number of elephants divided in statistical effect for the step-selection function.**

| Effect on preferences | cost of locomotion | NDVI | distance to water |
|---|---|---|---|
| Negative ($p < 0.05$) | 148 | 0 | 64 |
| None ($p > 0.05$) | 9 | 11 | 80 |
| Positive ($p < 0.05$) | 0 | 146 | 13 |

**Table S4: Average and standard deviation of the variables included in the SFF across all elephants.**

| Variable | average | standard deviation |
|---|---|---|
| cost of locomotion (kcal) | 81.10 | 27.20 |
| NDVI (adimensional) | 0.08 | 0.04 |
| distance to water (m) | 7677 | 4508 |

Average and standard deviations were calculated for each individual separately from values of the used and unused steps of the step-selection function and then averaged across all individuals.

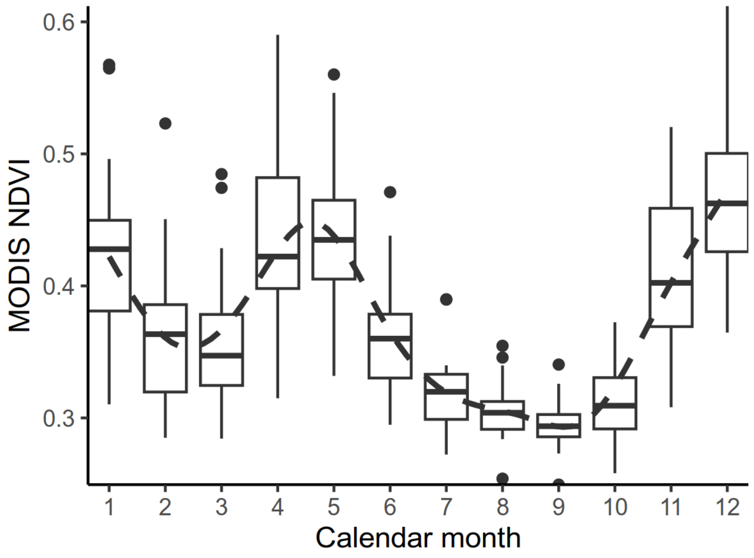


**Figure S1. NDVI monthly trend for the period 2001-2022 from MODIS.** Boxplots show the variability of monthly NDVI across years and the dashed black line the overall trend. The variability in monthly NDVI across years was noticable, but relatively small compared to the average yearly trend.

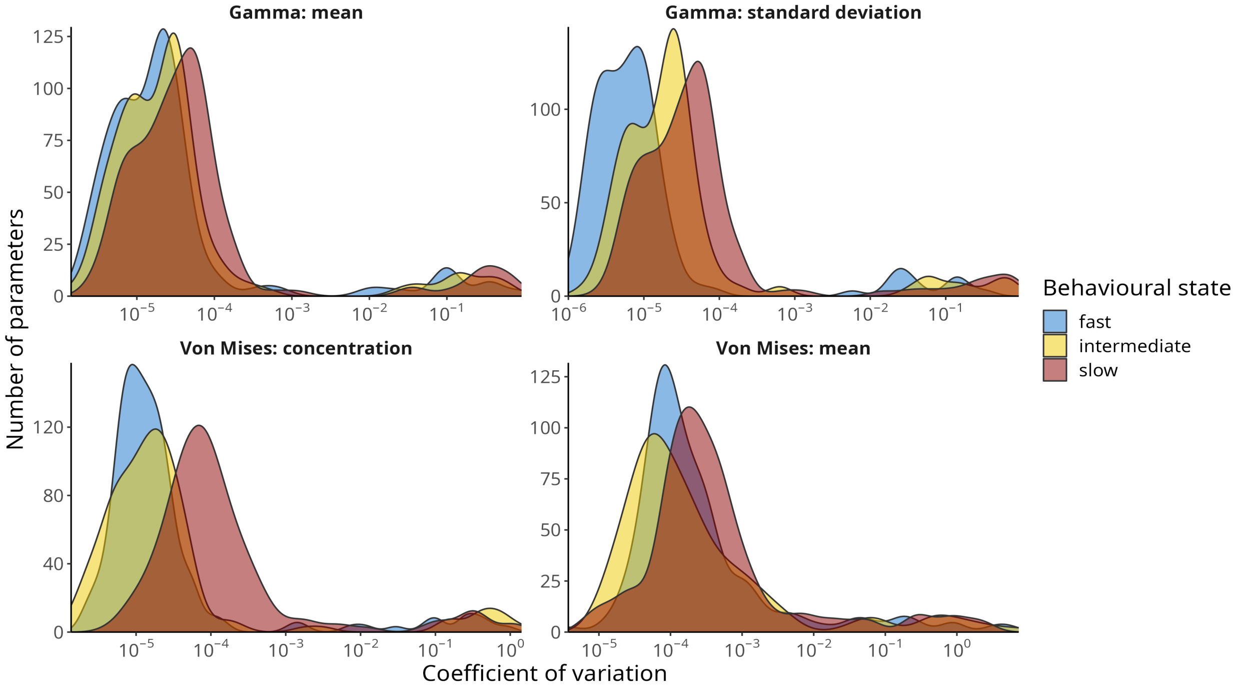


**Figure S2. Frequency distribution of the coefficient of variation of the Hidden Markov Models (HMMs) parameters.** The coefficient of variation quantifies how large was the standard deviation compared to the average fitted values and was calculated as abs(σ/μ) from the 10 HMM replicates for each individual. The colors show the three movement states, as fitted by the HMMs.

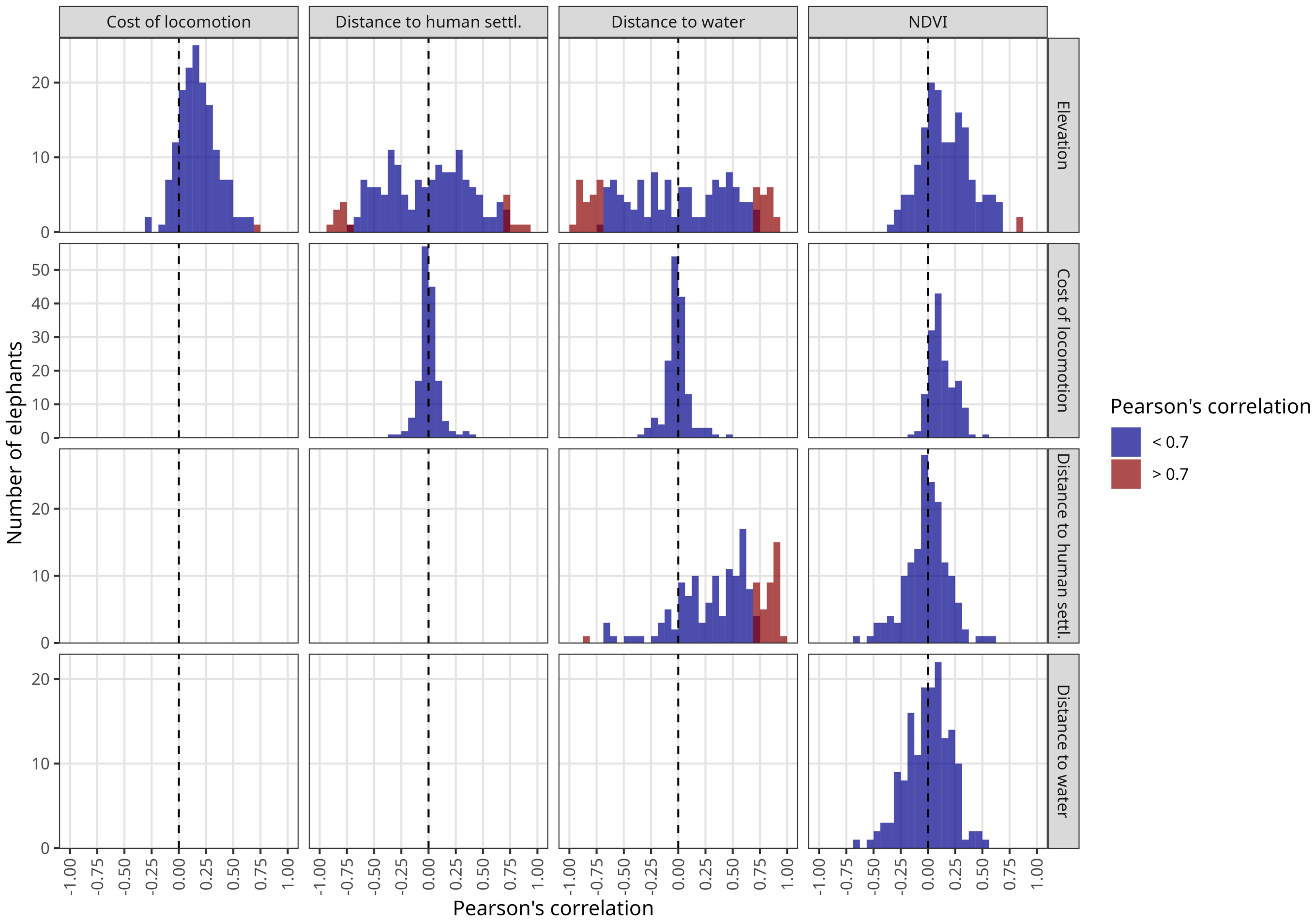


**Figure S3. Distribution of the Pearson's correlation coefficients between covariates among all elephants included in the study.** Histograms show the number of elephant with a given correlation coefficients, with colors indicating if the coefficient is higher than 0.7, a threshold commonly used to indicate high collinearity (Dormann et al, 2013).

# References

Dormann, C. F., Elith, J., Bacher, S., Buchmann, C., Carl, G., Carré, G., ... & Lautenbach, S. (2013). Collinearity: a review of methods to deal with it and a simulation study evaluating their performance. *Ecography*, *36*(1), 27-46.
